# Molecular detection and quantification of *Plasmodium vivax* DNA in blood pellet and plasma samples from patients in Senegal

**DOI:** 10.3389/fpara.2023.1149738

**Published:** 2023-04-24

**Authors:** Babacar Souleymane Sambe, Aissatou Diagne, Hélène Ataume Mawounge Diatta, Folly Mawulolo Gaba, Ibrahima Sarr, Arona Sabène Diatta, Serigne Ousmane Mbacké Diaw, Rokhaya Sané, Babacar Diouf, Inès Vigan-Womas, Babacar Mbengue, Makhtar Niang

**Affiliations:** ^1^ Institut Pasteur de Dakar, Pôle Immunophysiopathologie et Maladies Infectieuses, Dakar, Senegal; ^2^ Université Cheikh Anta Diop de Dakar, Service d’Immunologie, Faculté Médecine Pharmacie et Odonto-stomatologie, Dakar, Senegal

**Keywords:** malaria, *Plasmodium vivax*, diagnosis, blood pellet, plasma

## Abstract

**Background:**

The first discovery of *Plasmodium vivax* infections in Senegal used archived patients’ sera in place of blood pellet, the preferred specimen for the molecular diagnosis of *Plasmodium* species. The present study assessed the reliability of detecting *P. vivax* DNA in plasma in comparison to blood pellet from the same patient’s samples.

**Methods:**

A total of 616 blood samples obtained from febrile patients living in Kolda (2015 and 2020), Tambacounda (2017 and 2020), and Kedougou (2020) regions in Senegal, were first screened for *Plasmodium* species composition by 18S ssrRNA-based nested PCR. Paired blood pellets and plasma samples were selected from a subset of 50 *P. vivax*-positive patients matched by age and sex with 50 *P. vivax*-negative patients, and subjected to a cytochrome b-based qPCR to compare the detection and quantification of *P. vivax* genomic DNA between the two specimen types.

**Results and discussion:**

The study reports 1.8% and 14.77% of single and mixed *P. vivax* infections in the study population, and a high concordance (84%) between the qPCR detection of *P. vivax* genomic DNA from paired blood pellets and plasma samples. Importantly, all *P. vivax* negative samples from the blood pellets were also confirmed plasma-negative, and parasitaemia in blood pellets was higher compared to plasma samples. The results support investigations of *P. vivax* infections in archived sera or plasma collections with a high degree of confidence to generate additional data on the neglected *P. vivax* malaria, and ultimately guide strategies to control the disease.

## Background

Malaria remains a serious public health concern across the globe and is caused by infectious agents belonging to the genus *Plasmodium* of which five species (*P. falciparum, P. vivax, P. ovale, P. malariae* and *P. knowlesi*) are known to commonly infect humans (Hommel, 2007; Servonnet et al., 2012; WHO, 2020).

Capillary and venous blood are the usual biological materials for the detection of malaria parasites in human using light microscopy (LM) and/or Rapid Diagnostic Test (RDT) when the parasitaemia is high enough. While both diagnostic methods performed sufficiently well for diagnosing patients with acute symptomatic malaria, both methods could miss significant portions of low parasitemic individuals due to their detection limit of ~50 parasites/µl (Okell et al., 2009; Bousema et al., 2014). The limited sensitivity and specificity of both RDT and LM, and the lack of very experienced microscopists to distinguish between *Plasmodium* species may lead to poor malaria parasite diagnosis, and an underestimation of the true prevalence of non-*falciparum* parasites that are usually present at very low parasitaemia and in co-infections with the major *P. falciparum* species.

Various molecular assays have evolved as alternative diagnostics able to overcome the shortcomings of LM and RDT due to their significant improved sensitivity and specificity for the diagnosis of non-*falciparum* species. These molecular tests have been generally performed using DNA or RNA extracted from whole blood, the biological material of choice for malaria diagnosis. However, the detection of *P. falciparum* and *P. vivax* DNA in serum and plasma samples of confirmed *Plasmodium*-infected patients has opened up new perspectives for retrospective diagnosis of malaria infection and its potential association with other pathogens using archived sera or plasma specimen (Gal et al., 2001; Bharti et al., 2007; Niang et al., 2015; Niang et al., 2017; Niang et al., 2018).

In Senegal, *P. vivax* infections were first detected in long-term archived sera samples from febrile patients, which were initially collected for arbovirus surveillance in Kedougou, south-east Senegal (Niang et al., 2015). However, it is necessary to ensure the reliability of the diagnosis of plasmodial species using plasma or serum samples in order to better estimate the opportunities and limitations of this method. This study evaluated the detectability and quantification of *P. vivax* gDNA in blood pellets and plasma samples from febrile individuals.

## Material and methods

### Samples origin and types

The samples used in this study were obtained from 616 patients who consulted in healthcare facilities in Kedougou, Kolda, and Tambacounda, the three regions of Senegal with the highest incidence of malaria in 2021 (PNLP, 2022). Patients were recruited as part of a molecular survey of non-*falciparum* species infections, were aged between 0.5 to 83 years old, had fever (axillary temperature >37.5°C) and one or more symptoms suggestive of malaria disease (headache, nausea, dizziness, chills, fatigue, ….), and consented to be included in the study. For each participating patient, venous blood was collected in an EDTA tube and processed to separate plasma from the red blood cells (RBC) pellets, which were both stored at -20°C until used. The study objectives, benefits and risks were explained in French language or local dialects to all participants before inclusion. Written informed consent was obtained from all adult participants and from the parents, or legal guardians of children. The study was examined and approved by the Senegalese National Health Research Committee under the references 0081MSP/DS/CNRS and 00185 MSAS/CNERS/SP.

### Qualitative detection of species-specific *Plasmodium* parasite DNA from blood pellets

Two hundred microliters aliquots of frozen pellets were brought to room temperature and subjected to genomic DNA (gDNA) isolation using QIamp DNA Blood Mini Kit (Qiagen, Hilden, Germany). Elution of gDNA from pellet was performed on a volume of 100 µl. The qualitative detection of *Plasmodium* parasite DNA was based on nested PCR with primers targeting the *Plasmodium* spp. 18S small sub-unit ribosomal RNA (18S ssrRNA) gene as described previously Snounou & Singh (Snounou and Singh, 2002). Nested PCR results were scored as categorical variable (presence vs. absence of amplification).

### Detection of *Plasmodium vivax* DNA from plasma samples

Samples from 100 individuals with available paired plasma and RBC pellets were selected to compare the detection of *P. vivax* gDNA between the two biological materials. The samples were equally divided into two groups matched by age and sex. The first group included samples (DNA from RBC pellets and plasma) from 50 individuals who were positive for *P. vivax* by nested PCR from the RBC pellets (RBC/Pv+ group), while the second group were negative for *P. vivax* by using the same assay and biological material (RBC/Pv- group). Parasite gDNA from plasma samples was isolated using PureLink™ Genomic DNA Mini Kit (Thermo Fisher Scientific, USA) by strictly following the manufacturer’s instructions. DNA extraction from plasma was performed from an initial volume of 100 µl and final elution was done 50 µl volume.

### Quantification of *Plasmodium vivax* with reference reagent

#### Standard curve assay

The WHO international standard *Plasmodium vivax 19/116* (Pv19/116) standard for *Plasmodium vivax* antigen (LDH), obtained from the National Institute for Biological Standards and Control (NIBSC, Hertfordshire, United Kingdom, NIBSC code: 19/116) (Olomu et al., 2020) was used as the calibration reference reagent for the *P. vivax* detection and quantification assays, similar to that of *P. falciparum* (Padley et al., 2008). The Pv19/116 standard was made from *P. vivax* infected donors, and was provided as lyophilized RBC lysates in a vial at a concentration of 1000 IU of PvLDH. Following NIBSC recommendations, the lyophilized material was reconstituted within the vial by adding 250 µl of O+-type whole blood diluent from a healthy donor leading to a final concentration of 1000 IU/250 µl or 4.00 IU/µl. From the reconstituted Pv19/116 standard, 12 dilution series from 8.00 X 10^-01^ to 4.88 X 10^-05^ IU/µl were prepared using the same blood diluent and their resulting gDNA were used to generate a relative standard curve for the quantification of *P. vivax* DNA in clinical samples. The parasitemia-equivalent is therefore estimated using this standard curve.

#### Quantitative PCR assay

The standard curve calibration, molecular detection and quantification were performed by qPCR using primers targeting the *Plasmodium cytochrome b* gene. The amplification was done with the primers Pv_RTPCR previously described by Canier et al. (Canier et al., 2013) The reaction mixture is obtained as follows: 4 µl of Evagreen qPCR Mix plus (5X concentration), 0.5 µl of each forward and reverse primers at 10 µM concentration and 5 µl of DNA template, and 10 µl of H20 for a final volume of 20 µl. The assay parameters are next: 95°C 15 min, 95°C-10 s/62°C-20 s/72°C-20 sec to 40 cycles, 95°C-1 min, 40°C-1 min. A melt curve analysis followed all real-time PCR assay, according to the following parameters: from 65 to 90°C increment 0.2°C for 0.05 s. For samples that are positive, the temperature melt peak is between 77.40-77.80°C. Five replicates of each dilution were prepared to assess the assay reproducibility. A standard curve with a coefficient of determination of 98.4% was obtained. After the standard curve optimisation, three concentrations (8.00 X 10^-01^, 2.00 X 10^-01^, and 5.00 X 10^-02^ IU/µl) were selected as positive controls.

For quantification of DNA from RBC or plasma, the qPCR protocol used is strictly identical to that used to generate the standard curve. Each of the samples was run once except for the discordant ones for which the quantification has been done twice.

### Statistical analyses

Statistical analyses were performed using R software (R Core Team, 2021). The means and standard errors of the parasitaemias were calculated according to the biological material and the sampling period. The significance of the differences in mean parasite DNA concentration between the different groups was assessed with Mann-Whitney-Wilcoxon ranking test. The comparison of the diagnostic results from the two specimen types (blood pellets vs plasma) was performed by calculating the percentage of concordant PCR results between the two specimen types and Kendall’s correlation coefficient, tau (τ) was used to evaluate the degree of association. Significance level of statistical analysis was set at 5%.

## Results

### Characteristics of the study population

Demographic characteristics of the 616 patients whose blood samples screened for the presence of *Plasmodium* species parasite DNA were summarized in **Table 1**. In total, 178 patients were recruited in Kolda in 2015 (n = 80) and 2020 (n = 98), 358 patients in Tambacounda in 2017 (n = 137) and 2020 (n = 221), and 80 patients in Kedougou in 2020 (**Table 1**).

**Table 1:** Demographic characteristics of the study population.

Region Number	Kolda n = 178		Tambacounda n = 358		Kedougou n = 80
Year Number	2015 n = 80	2020 n = 98	2017 n = 137	2020 n = 221	2020 n = 80
Age (years)					
NA	0	3	79	0	0
Mean (SE)	24.64 (2.48)	22.31 (2.00)	10.37 (1.04)	20.36 (1.19)	21.54 (1.68)
Median	18	15	6	13	17.5
Range	0.91 - 77	0 - 83	0.5 – 63	0.13 - 78	1.04 - 70
Age groups (%, n)					
NA	0	3	79	0	0
0-5 years	30	18.95	37.93	21.72	3.75
5-15 years	11.25	33.68	44.83	32.13	36.25
+ 15 years	58.75	47.37	17.24	46.15	60
Gender (%, n)					
NA	0	0	77	0	0
Female	47.5	39.8	41.67	45.25	52.5
Male	52.5	60.2	58.33	54.75	47.5
Sex ratio (M/F)	1.11	1.51	1.4	1.21	0.9

n, Number of individuals; SE, Standard Error; NA, Not Available.

The age of the patients ranged from 0.5 to 83 years old and most of them (45%) originated from Tambacounda region (**Table 1**). The mean age of patients was around 20 years, but patients recruited in 2017 in Tambacounda were younger (10.37 ± 1.04 years). Male patients represented more than 50% except in patients enrolled in 2020 in Kedougou.

### Prevalence of *Plasmodium* species in blood samples

**Figure 1:**
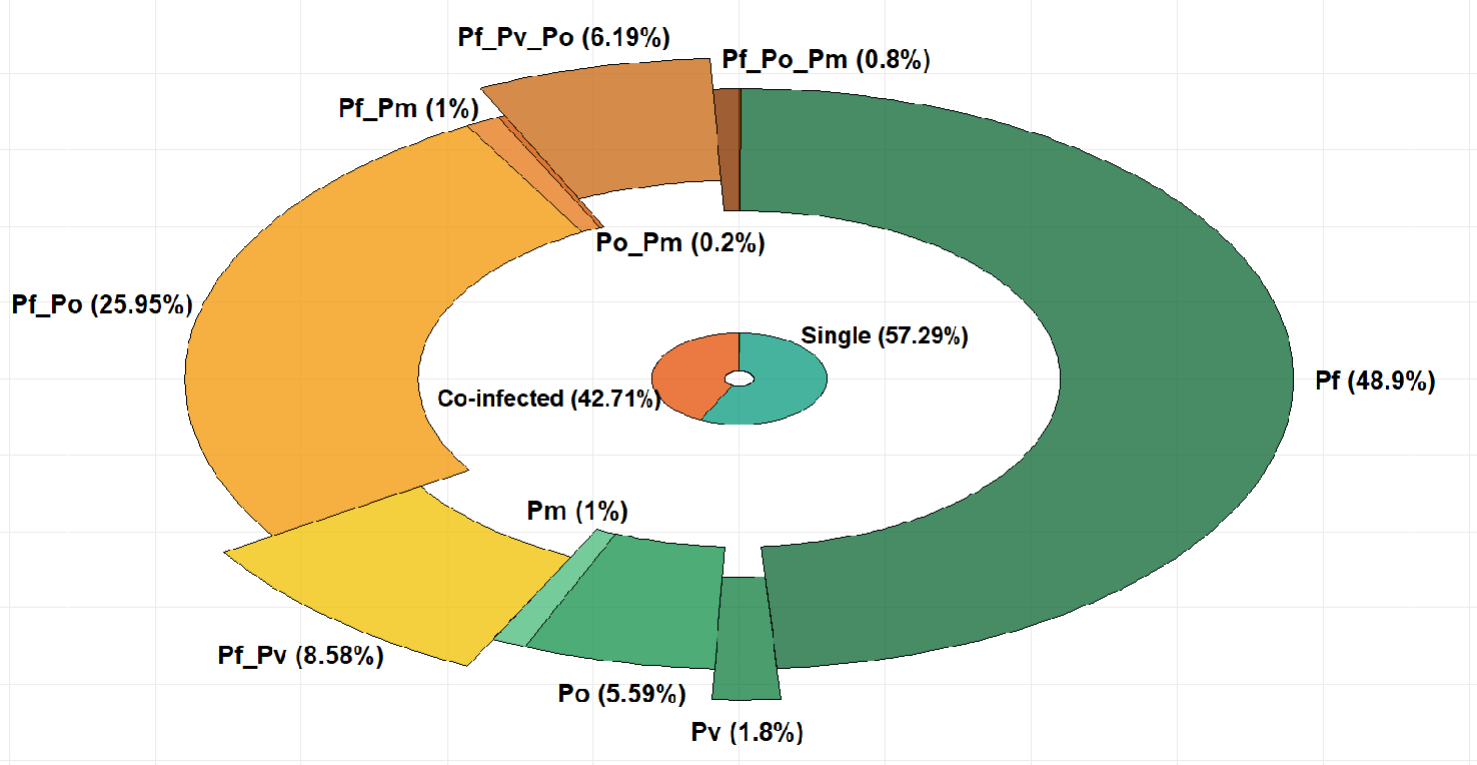
Proportions (%) of single and mixed *Plasmodium* species infections in the study population. (Pf, *P. falciparum*; Pv, *P. vivax*; Po, *P. ovale*;Pm, *P. malariae*).

Genomic DNA of the four *Plasmodium* species *P. falciparum, P. ovale*, *P. vivax*, and *P. malariae* was amplified by nested PCR at various proportions, either as single (57.29%) or mixed (42.71%) *Plasmodium* species infections (**Figure 1**). *P. falciparum* mono-infections account for the majority of infections and were detected in 48.9% of samples, while non-*falciparum* species mono-infections were detected in 1.8%, 1%, and 5.59% of samples for *P. vivax*, *P. malariae*, and *P. ovale*, respectively (**Figure 1**).

Among the mixed *Plasmodium* species infections, dual *P. vivax/P*. *falciparum* infections were found in 8.58% of samples while triple *P. vivax/P. falciparum/P. ovale* infections were present in 6.19% of samples (**Figure 1**). The number of infections in each category is provided as **Supplemental Table 1**.

### Details of patients selected for the detection of *P. vivax* in blood pellet and plasma samples

The demographics data of the 100 patients whose samples were selected and tested to compare *P. vivax* detection in blood pellet and plasma samples are summarized in **Table 2**. The M/F sex ratios were 1.17 and 1.42 whilst the mean age was 14.19 ± 1.92 and 13.76 ± 2.12 years for the *P. vivax*-negative and positive patients respectively (**Table 2**).

**Table 2:** Baseline characteristics of patients’ samples selected to compare *P. vivax* detection and parasitaemia in paired RBC pellet and plasma.

Group Number	RBC/Pv-n = 50	RBC/Pv+n = 50
Region (year)		
NA	0	0
Kolda (2020)	8	1
Tambacounda (2017)	11	38
Tambacounda (2020)	12	4
Kedougou (2020)	19	7
Age (years)		
NA	0	5
Mean (SE)	14.19 (1.92)	13.76 (2.12)
Median	10	8
Range	0.5 – 61	1 - 63
Age groups (%)		
NA	0	5
0-5 years	26	26.67
5-15 years	40	51.11
+ 15 years	34	22.22
Gender (%)		
NA	0	4
Female	46	41.3
Male	54	58.7
Sex ratio (M/F)	1.17	1.42

n, Number of individuals; SE, Standard Error; NA, Not Available; RBC, red blood cells.

**Figure 2:**
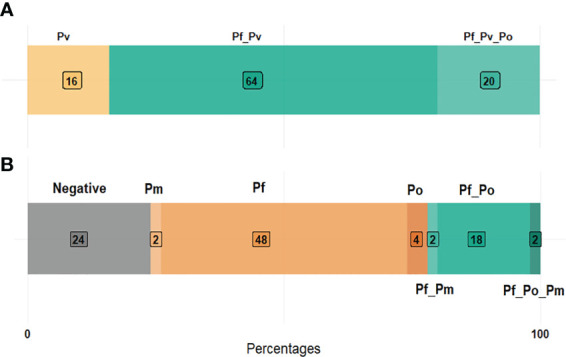
*Plasmodium* species composition in *P. vivax* positive **(A)** and negative **(B)** samples. (Pf, *Plasmodium falciparum*; Pv, *P. vivax*; Po, *P. ovale*; Pm, *P. malariae*).

Of the 50 *P. vivax* positive samples (**Supplemental Table 2**), 16% (8/50) were single and 84% (42/50) were mixed infections composed by *P. vivax/P. falciparum* (64%, 32/50) and *P. vivax/P. falciparum/P. ovale* (20%, 10/50) infections (**Figure 2A**). The *P. vivax*-negative group was composed by 24% (12/50) of negative samples, 54% (27/50) of single non-*vivax* infections, and 22% (11/50) of mixed non-*vivax* infections (**Figure 2B**).

### Concordance analysis of *P. vivax* gDNA detection between blood pellet and plasma samples

**Figure 3:**
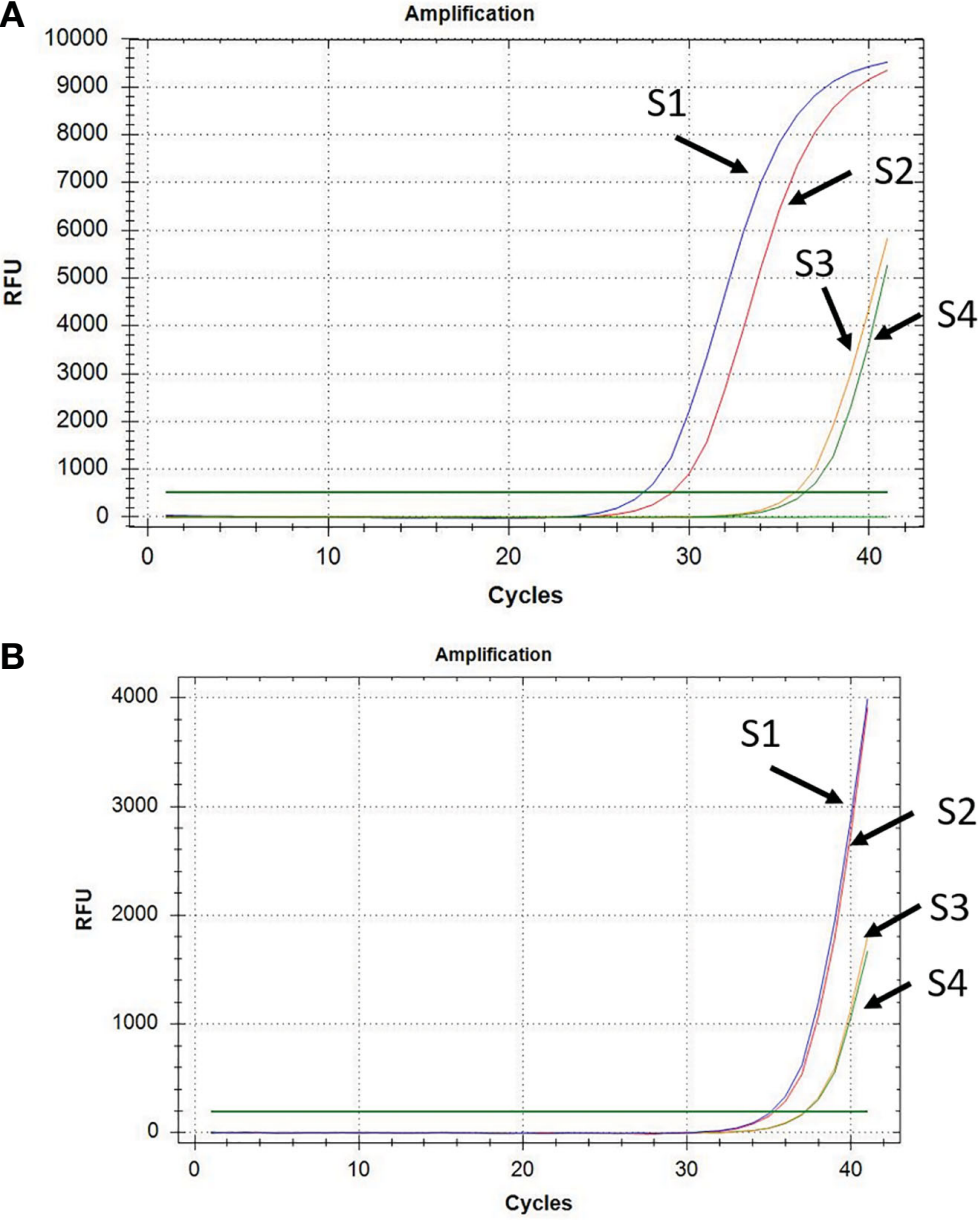
Amplification of *P. vivax* DNA from RBC pellet **(A)** and corresponding plasma **(B)** in a subset of four samples (S1 to S4).

The qPCR assays showed early amplification of *P. vivax* gDNA from RBC pellets (**Figure 3A**) compared to plasma (**Figure 3B**). In fact, usually, CT values for *P. vivax* DNA amplification from RBC pellets were lower and ranged between 28 to 37 cycles (**Figure 3A**), while *P. vivax* DNA amplification from plasma samples were observed at CT values between 36 to 39 cycles (**Figure 3B**).

**Figure 4:**
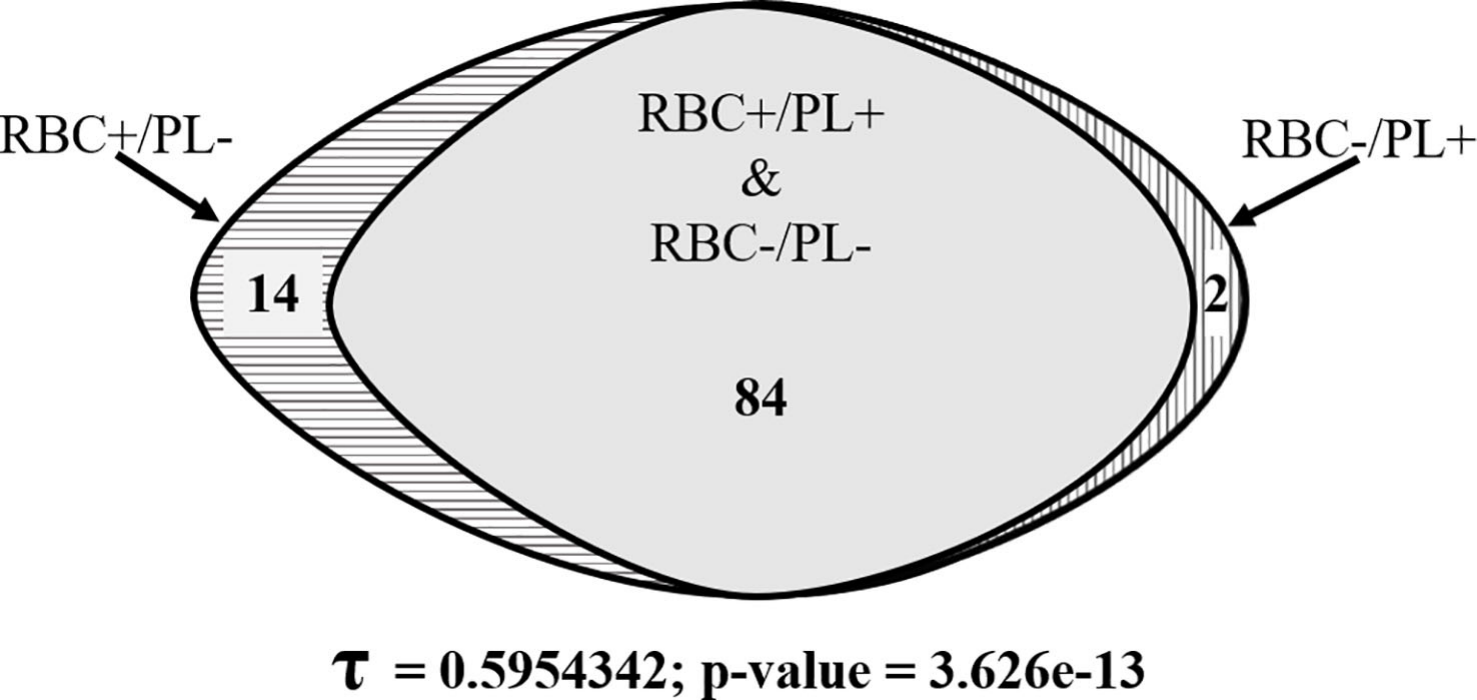
Venn diagram showing the degree of concordant and discordant qPCR detection of *P. vivax* gDNA from RBC pellets and plasma samples.

**Figure 4** shows an overall 84% concordance of PCR results and a significant Kendall’s correlation coefficient (τ = 0.59; p-value < 0.001) between results obtained with RBC pellets and plasma samples from the same patients. All samples negative for *P. vivax* in the RBC pellets were confirmed negative by using the paired plasma sample, whereas of the 50 RBC pellets that were positive for *P. vivax*, the positivity was confirmed in 34 paired plasma samples (**Figure 4**). The data also revealed 16% (16/100) of discordant PCR results between RBC pellets and plasma samples (**Figure 4**), corresponding to 2% RBC negative/plasma positive and 14% RBC positive/plasma negative samples, respectively (**Figure 4**). Discordant samples had very low parasitaemia ranging from 1.16 X 10^-04^ to 1.86 X 10^-03^ IU/µl, except for the sample collected in 2020 that had a parasitaemia of about 1.20 X 10^-02^ IU/µl.

### Comparison of *P. vivax* parasitaemia between RBC blood pellet and plasma samples

**Figure 5:**
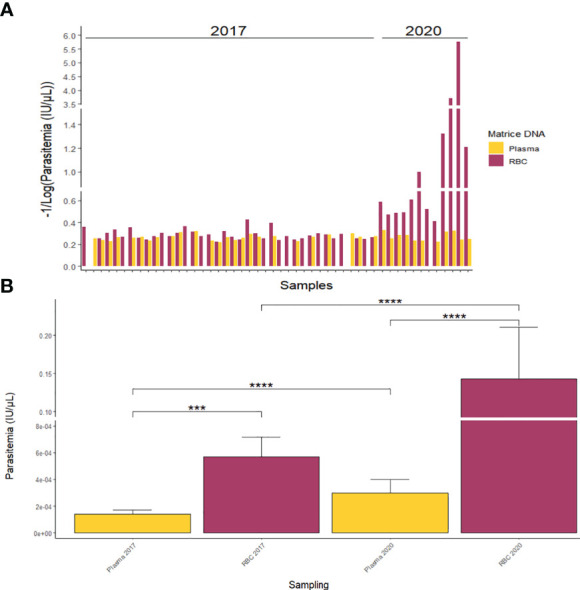
Quantification of *P. vivax* parasitaemia from RBC pellets and plasma at individual scale **(A)** and according to sampling year **(B)**. ***p-value < 0.001; ****p-value < 0.0001.

At the individual level, the comparison of *P. vivax* DNA detection in RBC pellets and plasma samples revealed a higher detection rate in the RBC pellets compared to the corresponding plasma along with a higher parasitaemia (expressed in IU/µl) in the RBC pellets’ samples (**Figure 5A**). Of the 38 *P. vivax* positive samples selected from Tambacounda in 2017 (**Table 2**), *P. vivax* gDNA was amplified in both RBC and plasma from 23 samples, of which only four samples had a higher plasma parasitaemia over the RBC pellets (**Figure 5A**). By contrast, *P. vivax* gDNA was amplified from both RBC pellet and plasma in 11 samples out of the 12 *P. vivax* positive samples of 2020 (**Figure 5A**), and the parasitaemia in all the 11 samples were significantly higher in the RBC pellets than the corresponding plasma (**Figure 5A**).

Globally, for both *P. vivax* positive samples collected in 2017 and 2020, the average parasitaemia was significantly (p-value < 0.001) higher in RBC pellets compared to plasma samples (**Figure 5B**). In fact, mean parasitaemias were respectively 1.41 X 10^-04^ ± 2.93 X 10^-05^ IU/µl and 5.71 X 10^-04^ ± 1.47 X 10^-04^ IU/µl in plasma and RBC pellets of 2017, and 2.98 X 10^-04^ ± 1.02 X 10^-04^ IU/µl and 1.43 X 10^-01^ ± 6.79 X 10^-02^ IU/µl in plasma and RBC pellets of 2020 (**Figure 5B**).

## Discussion

Global malaria elimination efforts are directed against *P. falciparum*, most likely because of the greater mortality rates associated with it, even though it is well acknowledged that *vivax* malaria is harder to prevent, diagnose, and treat. There exist a weaker knowledge base from which to start an effective global *vivax* malaria elimination program (Mueller et al., 2009). Sub-Saharan Africa populations have been presumably considered protected against endemic *P. vivax* malaria because of to the high prevalence of Duffy blood group negativity among its populations (Baird, 2022). Moreover, the lack of microscopists that are sufficiently trained to identify *P. vivax* parasites especially when they are in mixed infections with *P. falciparum* contributed to the limited diagnostic of *P. vivax* infections (Diallo et al., 2018). Finally, the strong tropism of *P. vivax* for the extravascular tissues of the bone marrow and the spleen would also explain the poor detection of *P. vivax* in the peripheral blood (Baird, 2022). In Senegal, the current literature highlights a low prevalence of *P. vivax*, but the screening of a large number of samples collected from different areas and periods has allowed to detect additional *P. vivax* positive samples and to enable the comparison of *P. vivax* detection from RBC pellets and plasma samples. The missing demographic information such as age and sex of few patients has no influence on the study findings and conclusion since no data was analyzed in relation with those parameters.

In sub-Saharan Africa, *P. vivax* is known to be present primarily as co-infections with *P. falciparum*, the dominant malaria species. The study findings demonstrated both single and mixed *P. vivax* infections in the study population. The results obtained in this study confirmed the previous reports on *P. vivax* circulation in Kedougou region (Niang et al., 2015; Niang et al., 2017), and indicated for the first time the presence of the parasite in samples collected from patients living in Kolda and Tambacounda regions. Since all patients included in this study had symptoms presumptive of malaria, the finding of patient’ samples mono-infected with *P. vivax* parasite raises concerns about its possible involvement in the disease burden in Senegal. Though no data is available in Senegal, the implication of *P. vivax* in clinical malaria and the disease severity has been described in some studies (Mahgoub et al., 2012; Nurleila et al., 2012), and it is generally accepted that control strategies for *P. falciparum* malaria are not adapted to *P. vivax* (Benavente et al., 2017; Benavente et al., 2021; Quaye et al., 2021).

The high proportions of concordant results in *P. vivax* parasite DNA detection from RBC pellets and their corresponding plasma further confirm the possibility to use plasma samples for the detection of *P. vivax*, though whole blood or RBC pellet remains the reference biological specimen for the molecular detection of *Plasmodium* parasites. This finding is important since it is widely admitted that plasma and sera samples are more often stored in biobanks, which would then offer the possibility of retrospective analysis and surveillance of other pathogens. In fact, the retrospective molecular and serological screening of archived sera were pivotal respectively to the discoveries of *P. vivax* infections and zika virus circulation in Kedougou region and Dielmo village in Senegal (Niang et al., 2015; Niang et al., 2017; Ba et al., 2018; Niang et al., 2018). The archiving and reuse of stored sera and plasma samples is of great interest in this particular worldwide context characterized by the emergence and re-emergence of new pathogens of major public health threat, as it may allow a rapid retrospective surveillance of circulating pathogens.

For the 14 samples (13 in 2017 and 1 in 2020) that were RBC-positive and plasma-negative for *P. vivax*, the absence of amplified *P. vivax* gDNA could be due to either a very low-level of genomic material below the detection level of our qPCR assay, a technical issue during the qPCR assay or the poor stability of preserved gDNA. The main issue in this study was the finding of only two samples that were *P. vivax* positive by nested-PCR with the RBC pellets-derived gDNA and by qPCR with plasma-derived gDNA, but were negative by qPCR with RBC-derived gDNA. Some technical issues during qPCR on RBC such as suboptimal cycling, insufficient or poor quality template, or pipetting error could explain these results.

However, the relative weakness of the sampling did not allow a more in-depth assessment of the impact of parameters such as the duration and conditions of sample’ storage. Also, this sampling does not allow the precise estimation of performance parameters such as sensitivity, specificity or threshold of detectability of *P. vivax* in plasma. This is an important area of research that need to be investigated in order to better understand the limits of the use of plasma in the detection of malaria parasite species.

Despite the effort to assess sufficient samples in the comparison analysis, the number of samples tested in this study could still be perceived as a limitation. However, the high degree of concordance between PCR results for the matched RBC pellet and plasma samples support the study conclusion, and demonstrate that our finding of a sizeable presence of *P. vivax* in the study areas are not due to laboratory error such as contamination of the nested PCR. The generation of further evidence for the use of plasma in the diagnosis of *Plasmodium* species infections would necessitate studies that would involve large numbers of symptomatic and parasitaemic patients that are followed-up over several periods. In addition, a quantitative study comparing the detection of *P. vivax* from patient’s sera or plasma during the course of the disease is necessary to better appreciate the clinical usefulness of serum/plasma-derived DNA in the diagnosis of malaria infection.

## Conclusion

This study revealed the presence of *P. vivax* in the three regions of Senegal with the highest incidence of malaria, and the detection of *P. vivax* gDNA in blood pellets and plasma samples from the same patients. The concordant PCR results obtained with the two specimen types were high, and the findings have important implications in the current context of global emergence of pathogens of public health concern as they encourage the retrospective surveillance of the circulating pathogens in specimen banks archived from cross-sectional or long-term follow-up studies.

## Data availability statement

The datasets supporting the conclusions of this article are included within the article. The data supporting the conclusions of this article can be made available by the authors under reasonable request.

## Ethics statement

The studies involving human participants were reviewed and approved by National Ethical Committee for Research in Health of Senegal. Written informed consent to participate in this study was provided by the participants’ legal guardian/next of kin.

## Author contributions

BSS, BM, IV-W, and MN conceived the study. BSS, AD, HAMD, FMG, IS, AD, SOMD, and RS performed the experiments. BSS, BD, AD, ASD, IS, BD and MN collected the samples. BSS, AD and MN analyzed the data and drafted the manuscript. BSS, BM, IV-W and MN supervised the study. All authors contributed to the article and approved the submitted version.
