# Supplementary material for: Molecular detection and quantification of Plasmodium vivax DNA in blood pellet and plasma samples from patients in Senegal
Source: Front Parasitol. 2023 Apr 24;2:1149738. doi: 10.3389/fpara.2023.1149738 (PMC11731676; doi:10.3389/fpara.2023.1149738)
Supplement: Supplementary file 1 [file Table_1.doc]

**Supplemental Table 1: Plasmodium composition of the study population**

| **Samples** | | **Kedougou** | **Kolda** | **Tambacounda** | **Total** |
| --- | --- | --- | --- | --- | --- |
| **Negative** | | 27 | 3 | 85 | 115 |
| **Single infected** | | **35** | **135** | **117** | **287** |
|  | **Pf** | 29 | 133 | 83 | 245 |
|  | **Pm** | 1 | 0 | 4 | 5 |
|  | **Po** | 1 | 2 | 25 | 28 |
|  | **Pv** | 4 | 0 | 5 | 9 |
| **Mixed** | | **18** | **40** | **156** | **214** |
|  | **Pf_Pm** | 1 | 1 | 3 | 5 |
|  | **Pf_Po** | 14 | 20 | 96 | 130 |
|  | **Pf_Pv** | 3 | 2 | 38 | 43 |
|  | **Po_Pm** | 0 |  | 1 | 1 |
|  | **Pf_Pv_Po** | 0 | 17 | 14 | 31 |
|  | **Pf_Po_Pm** | 0 | 0 | 4 | 4 |
| **Total** | | **80** | **178** | **358** | **616** |
