# Supplementary material for: Molecular detection and quantification of Plasmodium vivax DNA in blood pellet and plasma samples from patients in Senegal
Source: Front Parasitol. 2023 Apr 24;2:1149738. doi: 10.3389/fpara.2023.1149738 (PMC11731676; doi:10.3389/fpara.2023.1149738)
Supplement: Supplementary file 2 [file Table_2.doc]

**Supplemental Table 1: Plasmodium composition of the samples selected f**or the detection of vivax in blood pellet and plasma

| **Samples** |  | **RBC/Pv- group** | **RBC/Pv+ group** | **Total** |
| --- | --- | --- | --- | --- |
| Negative | | 12 | 0 | 12 |
| **Single** | | **27** | **8** | **35** |
|  | Pf | 24 | 0 | 24 |
|  | Pm | 1 | 0 | 1 |
|  | Po | 2 | 0 | 2 |
|  | Pv | 0 | 8 | 8 |
| **Mixed** | | **11** | **42** | **53** |
|  | Pf_Pm | 1 | 0 | 1 |
|  | Pf_Po | 9 | 0 | 9 |
|  | Pf_Po_Pm | 1 | 0 | 1 |
|  | Pf_Pv | 0 | 32 | 32 |
|  | Pf_Pv_Po | 0 | 10 | 10 |
| **Total** | | **50** | **50** | **100** |
